# Prediction of carbon dioxide solubility in sugar-water-alcohol solutions at high pressure for application to sparkling drinks

**DOI:** 10.1038/s41598-025-16266-0

**Published:** 2026-06-02

**Authors:** Shaker Al-Hasnaawei, Khalid Mujasam Batoo, Hardik Doshi, Anupam Yadav, R. Roopashree, Aditya Kashyap, Karthikeyan Jayabalan, Subhashree Ray, Ahmed Ahmed Ibrahim

**Affiliations:** 1 College of pharmacy, The Islamic University, Najaf, Iraq; 2 Independent Researcher, Riyadh, Saudi Arabia; 3 Independent Researcher, Rajkot, India; 4 Department of Computer Engineering and Application, GLA University, Mathura, 281406 India; 5 Department of Chemistry and Biochemistry, School of Sciences, Jain (Deemed to be University), Bangalore, Karnataka India; 6 Centre for Research Impact & Outcome, Chitkara University Institute of Engineering and Technology, Chitkara University, Rajpura, 140401 Punjab India; 7 Department of Chemistry, Sathyabama Institute of Science and Technology, Chennai, Tamil Nadu India; 8 Department of Biochemistry, IMS and SUM Hospital, Siksha ’O’ Anusandhan (Deemed to be University), Bhubaneswar, 751003 Odisha India; 9 Department of Physics and Astronomy, King Saud University, P. O. Box-2455, Riyadh, 11451 Saudi Arabia

**Keywords:** Sugar, PHSC, Solubility, Equation of state, Juice, Supercritical, Carbon dioxide, Chemical engineering, Chemical engineering, Physical chemistry

## Abstract

In the beverage industry, the CO_2_ pressure and dissolved CO_2_ reached in their packaged beverage play an important role. In this regard, the work aims to develop a thermodynamic model for the prediction of the phase equilibrium behavior of quaternary CO_2_–sugar–water-alcohol solutions up to high pressures. The Perturbed Hard Sphere Chain (PHSC) equation of state (EoS) has been used to predict the solubility of CO_2_ in glucose-water-alcohol solutions. Glucose molecules have been modeled as a chain-like molecule with ten association sites. Cross-association between CO_2_, glucose, and water molecules has been considered. Temperature-dependent binary interaction parameter (BIP) has been considered to improve the model performance. The solubility of glucose in water and ethanol has been estimated at various temperatures. As well, the vapor-liquid equilibrium behavior of the ternary CO_2_-glucose-water system has been predicted. The results show that the model can predict the solubility of CO_2_ in a glucose-water mixture satisfactory. In some sparking drinks, ethanol is widely used. In this regard, the prediction of CO_2_ solubility in the water-ethanol-glucose mixture has been investigated. The vapor-liquid equilibrium of the quaternary CO_2_-water-ethanol-glucose system has been predicted up to 30 MPa without using any additional adjustable parameter. The results show that, the deviation between model and experimental data in liquid and vapor phases increases by increasing ethanol concentration. The proposed model can be used for the prediction of CO_2_ solubility and equilibrium compositions of quaternary CO_2_-water-ethanol-glucose system over a wide range of pressures, temperatures, and sugar (or ethanol) concentrations.

## Introduction

High-pressure carbon dioxide (HPCD) is a non-thermal pasteurization technique that utilizes elevated levels of carbon dioxide under pressure to inactivate microorganisms in food products. This method has gained attention due to its effectiveness in extending the shelf life of various foods while preserving their sensory and nutritional qualities. HPCD has demonstrated effectiveness against various bacteria, yeast, and molds, making it suitable for a range of food products. Since it is a non-thermal process, the food maintains its freshness, flavor, and nutritional content better than heat treatments. By inactivating spoilage microorganisms, HPCD can significantly extend the shelf life of products like dairy, juices, sauces, and ready-to-eat meals. The antimicrobial effects of HPCD can reduce or eliminate the need for chemical preservatives. Fruit juices and carbonated drinks can benefit from HPCD, as they help eliminate pathogens while maintaining taste and freshness. Milk and cheese can undergo HPCD to improve safety and shelf life without compromising quality. Pre-packaged meals can be treated with HPCD to ensure safety and extend shelf life. In the context of sparkling drinks, HPCD involves dissolving carbon dioxide (CO_2_) into the beverage at high pressures, which not only carbonates the drink but also enhances its antimicrobial properties. The CO_2_ under high pressure exhibits antimicrobial effects, which can help in inactivating spoilage microorganisms and pathogens. The carbonic acid formed when CO_2_ dissolves in the beverage lowers the pH, creating an environment that is less favorable for microbial growth. HPCD can be used for fruit juices that are carbonated, targeting both microbial safety and flavor. High-pressure carbon dioxide treatment helps in maintaining the quality and safety of sparkling waters by minimizing microbial contamination. HPCD can also be adapted for soft drinks to improve safety and extend shelf life while ensuring optimal carbonation. By dissolving CO_2_ under high pressure, HPCD ensures a uniform distribution of bubbles throughout the liquid, leading to a consistent drinking experience. The antimicrobial properties of dissolved carbon dioxide help in inactivating spoilage microorganisms and pathogens in sparkling drinks. This is especially important for beverages that are minimally processed or have a shorter shelf life without preservatives. High-pressure CO_2_ can inhibit unwanted fermentation processes (e.g., by yeast) that may occur in sugar-containing drinks, helping to maintain the desired flavor profile and carbonation level. The pressure involved in the HPCD process can lead to considerations in packaging design. Containers must be capable of withstanding the high pressures involved, leading to innovations in packaging materials. Ongoing research and development may expand its applications and address any technical challenges associated with implementation.

Dohrn et al.^1^ measured the phase equilibrium data in the glucose-water-CO_2_ and glucose-water-ethanol-CO_2_ systems at temperatures up to 343 K and pressures up to 30 MPa. Their results show that CO_2_ solubility tends to decrease by increasing the glucose while adding ethanol resulting in increases in the solubility of glucose in the vapor phase. The results show that the solubility of glucose in pure supercritical CO_2_ is small. Calix et al.^2^ measured the CO_2_ solubility in pure water, ascorbic acid-sugars-water, citric acid-sugars-water, commercial orange juice, and apple juice. Their results showed that CO_2_ solubility in real juices was lower than in pure water. Sugars generally decrease CO_2_ solubility in water. This is because sugar molecules compete with CO_2_ molecules for interactions with water molecules. The higher the sugar concentration, the lower the CO_2_ solubility. This is a colligative property effect. High sugar content also increases the viscosity of the juice. Higher viscosity can slow down the rate at which CO_2_ dissolves or escapes, but it doesn’t necessarily change the equilibrium solubility as much as the competitive effect. In pure water, the addition of some acids can slightly increase CO_2_ solubility through a phenomenon called “salting-in.” This involves the ions from the acid interacting with water molecules, which may indirectly enhance CO_2_ interaction with water. However, this effect is usually small at the concentrations typically found in juices. In summary, while the acidic nature of juices might slightly enhance CO_2_ solubility, the high sugar content generally decreases it. The precise effect depends on the specific composition of the juice.

The amount of CO_2_ in the food plays an important role in to scale-up of laboratory experiments for commercial application of HPCD. Experimental measurements can be time-consuming, labor-intensive, and require specialized equipment (e.g., high-pressure cells, and analytical instruments). Each measurement only provides data for a single set of conditions. It’s often impractical to experimentally measure solubility across a wide range of temperatures, pressures, and compositions. The results of experimental measurement are only applicable to the specific system studied. Extrapolating to significantly different conditions or systems can be unreliable. Thermodynamic models can predict gas solubility under a wide range of conditions, including those that are difficult or impossible to measure experimentally. Once a model is developed and validated, it can be used to generate solubility data relatively quickly and inexpensively. As well, models can also be used to extrapolate beyond the range of experimental data or interpolate between data points. Thermodynamic models can be used to optimize processes that involve gas solubility, such as gas absorption, stripping, and extraction. On the other hand, thermodynamic models have some disadvantages. Thermodynamic models often require parameters that must be estimated from experimental data or other sources. The accuracy of the predictions depends on the accuracy of these parameters. Even well-validated models can have limitations and may not accurately predict gas solubility under all conditions, especially near critical points or in complex mixtures. In many cases, a combination of both experimental measurement and thermodynamic modeling is the best approach. Experimental data can be used to develop and validate thermodynamic models, which can then be used to predict gas solubility under conditions that are difficult or impossible to measure experimentally.

Thermodynamic properties and phase equilibrium calculations of sugar-containing systems can be modeled using the Equation of State (EoS) models or activity coefficient models^3–6^. The NRTL (NonRandom Two-Liquid)^7^ and UNIQUAC (UNIversal QUAsi-Chemical)^8^ models are the most important models of activity-based approach. Helmholtz free energy-based models such as Statistical Association Fluid Theory (SAFT)^9^ were widely used for the description of thermodynamic properties of sugar-containing systems. Abderafi and Bounahmidi used bubble temperature experimental data of aqueous sugar solutions to adjust binary interaction parameters for Peng and Robinson (PR), and Kesler and Lee equations of state, and NRTL activity coefficient model^10^. Peres and Macedo modified the combinatorial term of the existing UNIQUAC model and calibrated its parameters using the bubble temperature experimental data of aqueous sugar solutions^11^. The UNIFAC is the most popular model for calculating interactions among several components, including sugars^12^. UNIFAC has a more predictive character than it is based on the group contribution approach, which reduces the amount of experimental data required for parameter calculation. Gros and Dussap utilized the UNIFAC model to estimate water activity, osmotic pressure, melting point, pH, and acidity of bovine milk^13^. Abderafi and Bounahmidi utilized the PR EoS, for binary, ternary, and quaternary solutions of sugars-containing systems. They calculate the bubble temperature of beet and sugar cane juice^14^. Velezmoro et al. studied the water activity in binary and quaternary mixtures containing sucrose, glucose, fructose, and water using cubic EoS^15^. Wei Feng et al. used the SAFT EoS to estimate the solubility of D-fructose, D-glucose, and sucrose in water and alcohol^16^. C. Held et al. used the Perturbed-chain SAFT (PC-SAFT) EoS to calculate the solubility of glucose, fructose, fucose, xylose, maltose, galactose, mannitol, mannose, sorbitol, xylitol, lactose, trehalose, and sucrose in water and alcohol^17^. Lucas T. Paese et al. utilized the COnductor-like Screening MOdel (COSMO) model to predict bubble point pressure, bubble point temperature, water activity, freezing point depression and solubility of sugars in aqueous solutions^18^. They predicted industrial juice water activity, bubble point temperature, and freezing point depression. Their results show that, COSMO model results were in good agreement with experimental data. G. Ferrentino et al. measured and modeled the CO_2_ solubility in apple juices at high pressure^19^. They measured CO_2_ solubility in ternary mixtures of water-CO_2_-glucose and water-CO_2_-malic acid, and a quaternary system of water-CO_2_-malic acid-glucose at different concentrations of malic acid and glucose. They predicted the CO_2_ solubility in ternary and quaternary mixtures using a cubic-base EoS. Their results were in good agreement with experimental data.

The main goal of this study is the prediction of phase equilibrium behavior of CO_2_-sugar-ethanol-water systems up to high pressures for application to sparkling drinks. In this regard, a perturbation-based EoS has been considered to model molecular interactions between like and unlike molecules. In recent years, the Perturbed Hard-Sphere Chain (PHSC) EoS has been utilized to model the thermodynamic properties and phase equilibrium calculations of biodiesel^20^ amino acids^21^ electrolyte solutions^22^ hydrate formation^23^ and gas solubility in ionic liquids and electrolyte solutions^24,25^. Recently, A. H. Adhab et al. utilized the PHSC EoS to model the solubility of sugars in mixed solvents^26^. They studied thirteen sugars containing glucose, fructose, xylose, maltose, mannitol, mannose, sorbitol, xylitol, galactose, erythritol, maltitol, trehalose, and sucrose. They showed that, the PHSC EoS can be utilized as a robust thermodynamic model to predict the solubilities of sugars in mixed solvents^26^. The repulsive, dispersion, chain, and association interactions between all molecules in ternary and quaternary systems can be modeled using the PHSC EoS to predict the solubility of CO_2_ at high pressures. In this regard, the PHSC EoS has been utilized for the prediction of phase equilibrium calculations of ternary and quaternary aqueous solutions containing glucose and CO_2_.

## Thermodynamic modeling

### The PHSC EoS

The PHSC EoS provides a useful and relatively simple way to model the thermodynamic properties of fluids, particularly those containing chain-like molecules. They balance accuracy and computational efficiency, making them valuable tools for chemical engineers and scientists. The specific form of the PHSC EoS can vary depending on the approximations used for the chain and perturbation terms. The PHSC can provide reasonable accurate predictions of thermodynamic properties for a wide range of fluids, especially chain-like molecules. As well, PHSC is computationally faster than molecular simulations, making them suitable for process simulations and engineering applications. PHSC model is relatively simple to understand and implement compared to more complex molecular simulation techniques. The PHSC model was developed using the modified Chiew EoS^27,28^. The compressibility factor of the PHSC EoS is as follows:

1 $$\:Z={Z}^{PHSC}+{Z}^{assoc}$$

2 $$\:{\mathrm{Z}}^{PHSC}={Z}^{ref}+{Z}^{pert}$$

3 $$\:{Z}^{pert}=-\frac{\rho\:}{{k}_{b}T}\sum\:_{i,j}{x}_{i}{x}_{j}{r}_{i}{r}_{j}{a}_{ij}\left(T\right)$$

4 $$\:{Z}^{ref}={Z}^{HS}+{Z}^{chain}=1+\rho\:\sum\:_{i,j}{x}_{i}{x}_{j}{r}_{i}{r}_{j}{b}_{ij}\left(T\right){g}_{ij}^{hs}\left({d}_{ij}\right)-\sum\:_{i}{x}_{i}\left({r}_{i}-1\right)\left[{g}_{ij}^{hs}\left({d}_{ij}\right)-1\right]$$

$\:{Z}^{pert}$ is the perturbation term accounting for attractive forces. This is usually based on a mean-field approximation and involves parameters related to the intermolecular potential. $\:{Z}^{HS}$ is the compressibility factor of the hard-sphere reference fluid. $\:{Z}^{chain}$ is the contribution due to chain connectivity. This term accounts for the reduced entropy and free volume resulting from the bonding of the hard spheres into chains. It depends on the chain length (number of segments) and the packing fraction.

The parameters, *a(T)* and *b(T)* given by Song et al.^29^:

5 $${a_{ij}}(T)=\frac{{2\pi }}{3}{\sigma _{ij}}^{3}{\varepsilon _{ij}}{F_a}(\frac{{{k_B}T}}{{{\varepsilon _{ij}}}})$$

6 $${b_{ij}}(T)=\frac{{2\pi }}{3}{\sigma _{ij}}^{3}{F_b}(\frac{{{k_B}T}}{{{\varepsilon _{ij}}}})$$

For a mixture, classical combining rules are used for calculation of $\sigma _{{ij}}$ and $\varepsilon _{{ij}}$as follows:

7 $$\:{\sigma\:}_{ij}=\frac{\left({\sigma\:}_{ii}+{\sigma\:}_{jj}\right)}{2}(1-{l}_{ij})$$

8 $$\varepsilon _{{ij}} = \sqrt {\varepsilon _{{ii}} \varepsilon _{{jj}} } (1 - k_{{ij}} )$$

The Boublik–Mansoori–Carnahan–Starling equation is used for the radial distribution function of hard sphere mixtures^30^:

9 $$g_{{ij}} ^{{hs}} (d_{{ij}} ^{ + } ) = \frac{1}{{1 - \eta }} + \frac{3}{2}\frac{{\zeta _{{ij}} }}{{(1 - \eta )^{2} }} + \frac{1}{2}\frac{{\zeta _{{ij}} }}{{(1 - \eta )^{3} }}$$

$\:{Z}^{assoc}$ is defined based on the SAFT model introduced by Chapman et al.^9^:

10 $$\:{Z}^{assoc}=\rho\:\sum\:{x}_{i}\sum\:_{{A}_{i}}\left[\frac{1}{{X}^{{A}_{i}}}-\frac{1}{2}\right]\left(\frac{\partial\:{X}^{{A}_{i}}}{\partial\:{\rho\:}_{i}}\right)$$

11 $$\:{X}^{{A}_{i}}={\left[1+\rho\:\sum\:{x}_{i}\sum\:_{{B}_{j}}{X}^{{B}_{j}}{{\Delta\:}}^{{A}_{i}{B}_{j}}\right]}^{-1}$$

12 $$\:{{\Delta\:}}^{{A}_{i}{B}_{j}}={g}_{ij}^{hs}\left({d}_{ij}^{+}\right)\left[exp\left(\frac{{\epsilon\:}^{{A}_{i}{B}_{j}}}{{k}_{b}T}\right)-1\right]\left({\sigma\:}_{ij}^{3}{\kappa\:}^{{A}_{i}{B}_{j}}\right)$$

The cross association energy and volume are calculated according to the combining rules proposed by Wolbach and Sandler^31^:

13 $$\:{\epsilon\:}^{{A}_{i}{B}_{j}}=\frac{1}{2}\left({\epsilon\:}^{{A}_{i}{B}_{i}}+{\epsilon\:}^{{A}_{j}{B}_{j}}\right)$$

14 $$\:{\kappa\:}^{{A}_{i}{B}_{j}}=\sqrt{{\kappa\:}^{{A}_{i}{B}_{i}}{\kappa\:}^{{A}_{j}{B}_{j}}}\:{\left(\frac{\sqrt{{\sigma\:}_{i}{\sigma\:}_{j}}}{0.5\left({\sigma\:}_{i}{+\sigma\:}_{j}\right)}\right)}^{3}$$

The PHSC model was widely used for the prediction of thermodynamic properties of electrolytes, surfactants, and associating components^20,22,24,29,32,33^. PHSC EoS typically involves parameters that need to be determined by fitting to experimental data. These parameters are often related to the size and shape of the molecules. In the next section the model parameter estimation of the PHSC EoS has been studied.

## Results and discussion

### Model parameters

**Fig. 1:**
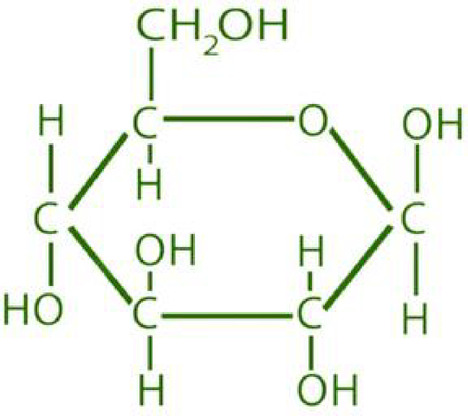
Molecular structure of Glucose molecule.

The pure model parameters have been obtained using vapor pressure saturated liquid density and Solid-Liquid equilibrium (SLE) calculations. The PHSC EoS have five model parameters containing segment number (r), segment diameter (σ), segment energy (ε), association energy (ε^AB^), and association volume ($\:{\kappa\:}^{AB}$). In the case of water, ethanol, and CO_2_, the pure model parameters were adjusted using experimental vapor pressure and saturated liquid experimental data. The previous works showed that, the PHSC^25^ and PC-SAFT^34^ performance can be improved by considering the association interaction between CO_2_ molecules (self-association), and cross-association between CO_2_ and solvents. In the case of sugars (glucose in this study), the model parameters were adjusted using SLE calculations^26^. In the case of water and alcohols two association sites (2B scheme) based on the Huang and Radosz association scheme have been considered^35^. Glucose molecule is modeled as an associating component with ten association sites (five donor and five acceptor sites). Glucose molecules contains five hydroxyl groups. For each hydroxyl group two association sites (a donor and an acceptor sites) have been considered; see Fig. 1.

The water model parameters were reported in reference^21,22,25^. D. Ramdan et al. considered 4 C scheme for CO_2_ molecules to predict the solubility of CO_2_ in electrolyte solutions^25^. Their results showed that the 4 C association scheme for CO_2_ molecules improves the model performance dramatically. In this work, the 4 C scheme has been considered for CO_2_ molecules. In the case of glucose pure parameters, A. H. Adhab et al. utilized the SLE experimental data to adjust the sugar parameters^26^. In this work, a BIP between water and glucose has been considered to improve the model performance. The pure model parameters and BIPs have been reported in Tables 1 and 2.

**Table 1:** Pure glucose, water, methanol, and CO_2_ parameters.

Sugars	Parameters						Ref.
	*r*(-)	$\:\sigma\:\left(\dot{\mathrm{A}}\right)$	$\:\epsilon\:/{k}_{B}\left(\mathrm{K}\right)$	$\:{\epsilon\:}^{ab}/{k}_{B}\left(\mathrm{K}\right)$	$\:{k}^{AB}$	*N* ^Assoc^	
Glucose	8.059	3.449	281.824	2500	0.03	10*	^26^
Water	1.405	3.043	460.52	1633.28	0.03804	2B	^25^
Ethanol	1.821	4.044	265.38	2644.31	0.01049	2B	^33^
CO_2_	4.781	0.2277	115.31	321.501	0.02220	4 C	^25^

* 5 donor and 5 acceptor association sites.

**Table 2:** The binary interaction parameter (*k*_*ij*_) between components. T is kelvin.

k_ij_				
	Glucose	Water	Ethanol	CO_2_
Glucose	0	0.000659T − 0.184092	-0.000555T + 0.178391	0
Water	0.000659T − 0.184092	0	0.0004T − 0.2086	− 0.0000071536T^2^ + 0.006370T- 1.31098
Ethanol	-0.000555T + 0.178391	0.0004T − 0.2086	0	-0.000595T + 0.356699
CO_2_	0	− 0.0000071536T^2^ + 0.006370T- 1.31098	-0.000595T + 0.356699	0

In this work, the BIPs between CO_2_-ethanol and glucose-ethanol have been adjusted using VLE data (refer to Sect. "Binary and ternary VLE calculations"). The BIPs of ethanol-water, CO_2_-water systems were adjusted in references^26^ and^25^, respectively. The results showed that, an additional BIP (*l*_*ij*_) is required to reduce the average error value of the model for CO_2_-H_2_O system^32^. Therefore, in addition to *k*_*ij*_, a *l*_*ij*_ between CO_2_ and water was adjusted to improve the model performance, especially in the vapor phase (*l*_*ij*_= -0.000003468T^2^ + 0.002203T- 0.16660). As shown in Tables 1 and 2, the temperature-dependent BIPs are required to correlate the binary VLE or SLE data over a wide range of temperatures and pressures. In some cases, quadratic equation has been utilized to improve the model performance. In this work the Shuffled Complex Evolution (SCE) method, often referred to as SCE has been used to adjust the model parameters^36^. It is a global optimization algorithm particularly effective for solving complex, non-linear, non-differentiable, and multimodal problems. In Sect. 3.2 the VLE of binary and ternary systems have been studied.

### Binary and ternary VLE calculations

The phase equilibria of CO_2_-containing systems are essential for understanding various natural and industrial processes, including carbon capture and storage (CCS), natural gas production, and the behavior of CO_2_ in geological formations. Phase diagrams illustrate the stability regions of different phases of CO_2_ based on temperature and pressure. In many natural and industrial processes, CO_2_ is in contact with water, leading to complex equilibria. High-pressure VLE data for CO_2_-alcohol systems is crucial for designing and optimizing processes like supercritical fluid extraction, CO_2_-based solvents, and chemical reactions in supercritical CO_2_. These systems often exhibit significant non-ideality, especially at higher pressures and near critical points.

Alcohols are polar molecules due to the hydroxyl (-OH) group, while CO_2_ is a non-polar molecule. This difference in polarity leads to significant deviations from ideal behavior, resulting in complex phase behavior. The strength of the interaction depends on the alcohol’s molecular structure (such as chain length or branching). The hydroxyl group in alcohols participates in strong hydrogen bonding, affecting both the vapor-liquid equilibrium and the solubility of CO_2_ in the liquid phase. This hydrogen bonding can be disrupted by CO_2_, impacting the overall system behavior. On the other hand, alcohols tend to self-associate through hydrogen bonding, forming clusters or dimers. This self-association competes with the CO_2_-alcohol interactions, influencing the phase behavior.

This makes accurate thermodynamic modeling challenging. The low molecular weight alcohols are among the most important compounds in separation processes. Fornari et al.^37^ Jennings et al.^38^ Staby and Mollerup^39^ Dohrn and Brunner^40^ suggested methods to measure the phase equilibria of CO_2_-containing systems.

**Fig. 2:**
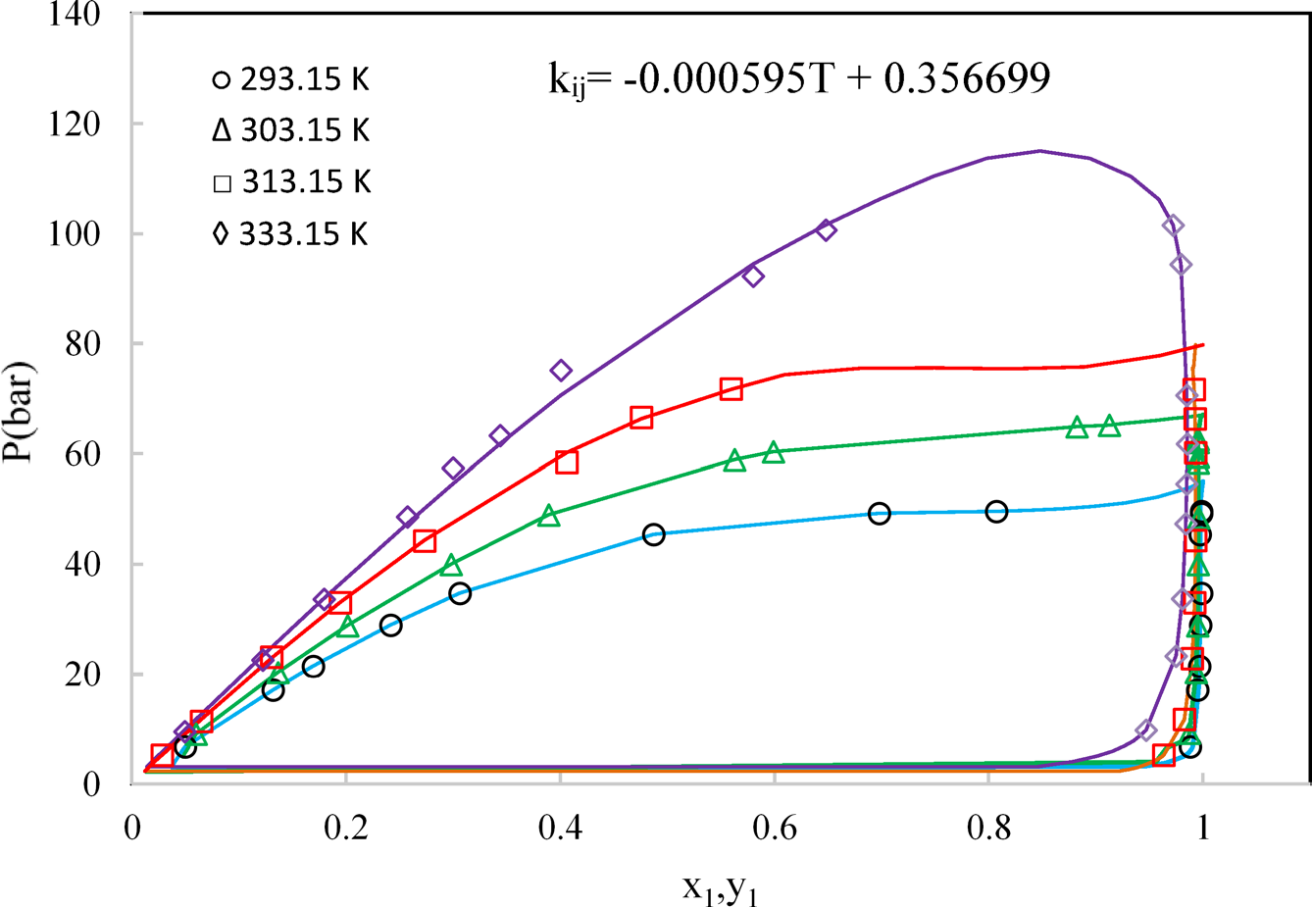
Comparison of literature data^41^ for the carbon dioxide (1) + ethanol (2) system and estimated with PHSC EoS.

In this study, the phase equilibrium of binary CO_2_-ethanol up to high pressure has been modeled using the PHSC EoS. A BIP between CO_2_ and ethanol has been adjusted using available experimental data; see Table 2. In Fig. 2, the VLE of the binary CO_2_-ethanol system has been depicted.

The results show that a temperature-dependent BIP is required to improve the model performance up to high pressures. The vapor and liquid phase compositions have been estimated using one temperature-dependent BIP. In this work, the cross-association between CO_2_ and ethanol has been considered similar to water-CO_2_ systems. Two BIPs (*k*_*ij*_ and *l*_*ij*_) have been adjusted to correlate the CO_2_ solubility in water at high pressures and temperatures. While the *l*_*ij*_ between CO_2_ and ethanol has been set to zero (*l*_*ij*_*=0.0*). The results show that, the PHSC EoS can estimate the solubility of CO_2_ in ethanol using one temperature-dependent BIP (*k*_*ij*_) accurately. The average relative deviation (ARD%) of total pressure for CO_2_-ethanol system has been obtained about 2.8%. The results indicated that a BIP is needed to calculate the vapor and liquid equilibrium compositions of CO_2_-ethanol system, accurately.

**Fig. 3:**
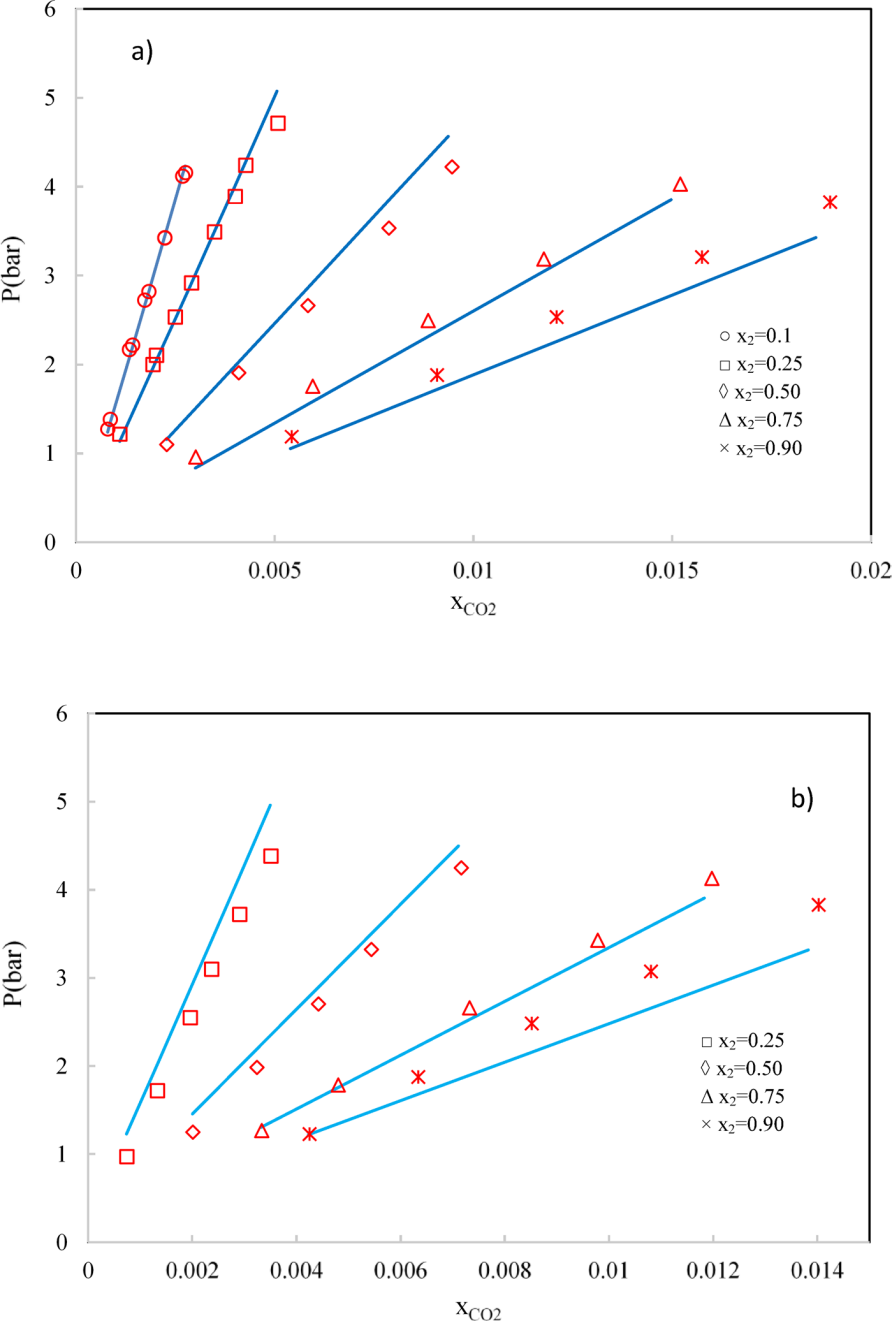
P–x diagram for carbon dioxide (1) in water (3)–ethanol (2) mixtures at (**a**) 298 K and (**b**) 323 K. Lines are model calculations and symbols are experimental data^42^. x_2_ refers to ethanol mole fraction based on CO_2_-free solutions.

In the case of ternary CO_2_-ethanol-H_2_O system, the BIPs between CO_2_-ethanol, ethanol-H_2_O, and CO_2_-H_2_O in Table 2 have been used to predict the solubility of CO_2_ in ethanol-water solvent without using any additional adjustable parameters. In Fig. 3, the solubility of CO_2_ in ethanol-H_2_O system has been depicted.

As shown in Fig. 3, the P-x diagram of the ternary CO_2_-H_2_O-ethanol system has been predicted at various ethanol concentrations and temperatures. As shown in Fig. 3a and b, the PHSC model can predict the solubility of CO_2_ in ethanol-water mixtures satisfactory. The average ARD% value of the PHSC EoS has been obtained at about 4.2%. The results are in good agreement with experimental data. It must be noted that the results are pure predictions because the VLE of the ternary system has been estimated without using any additional adjustable parameters.

In this study, the SLE calculation of ternary glucose-ethanol-water has been studied. In the case of SLE calculation, the composition of solute (glucose) in solvent must be calculated. The mole fraction of solute *i* in ethanol + water solvent has been calculated by:

15 $$\:-\mathrm{ln}\left({x}_{i}{\gamma\:}_{i}^{s\:}\right)=\frac{\varDelta\:{H}_{m}}{R}\left(\frac{1}{T}-\frac{1}{{T}_{m}}\right)+\frac{\varDelta\:{C}_{p}}{R}\mathrm{ln}\left(\frac{{T}_{m}}{T}\right)-\frac{\varDelta\:{C}_{p}}{R}(\frac{{T}_{m}}{T}-1)$$

where *x*_*i*_ and $\:{\gamma\:}_{i}^{s\:}$ are the mole fraction and the symmetric activity coefficient of the glucose in the liquid solvent. *∆H*_*m*_ stands for the melting enthalpy, *T*_*m*_ is the melting temperature of the glucose and *∆C*_*p*_ is the difference in solute heat capacity between liquid and solid at the melting point. The activity coefficient of component *i* is defined as follows:

16 $$\:{\gamma\:}_{i}^{s\:}=\frac{{\phi\:}_{i}(T,P,xi)}{{\phi\:}_{0i}(T,P,xi\to\:1)}$$

**Fig. 4:**
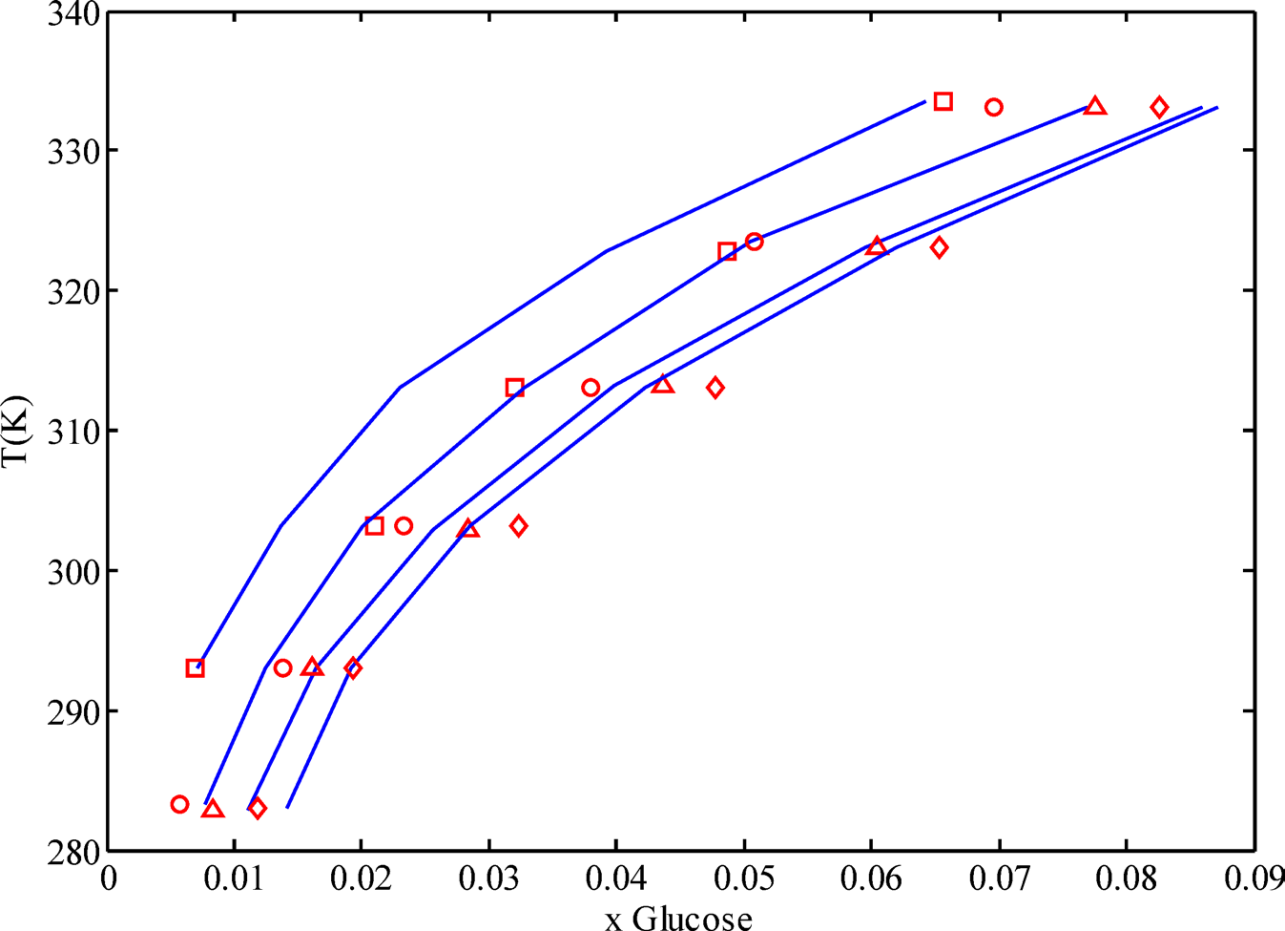
Glucose solubility in water + ethanol. Ethanol/water: Symbols ◊ (50 to 50) %; ∆ (60 to 40) ○ (70 to 30) %, □ (80 to 20) % are experimental data^46^ and lines refer to model predictions.

The melting temperature and enthalpy change are available in the literature^43–45^. In the case of glucose, melting temperature, melting enthalpy, and heat capacity difference are 423.2 K, 183 J/mol/K, and 42,432 J/mol, respectively. In Eq. (16), the fugacity of glucose in a mixture and pure state has been calculated by the PHSC EoS. In Fig. 4, the solubility of glucose in the ethanol + water mixture at four ethanol/water weight ratios has been predicted.

**Fig. 5:**
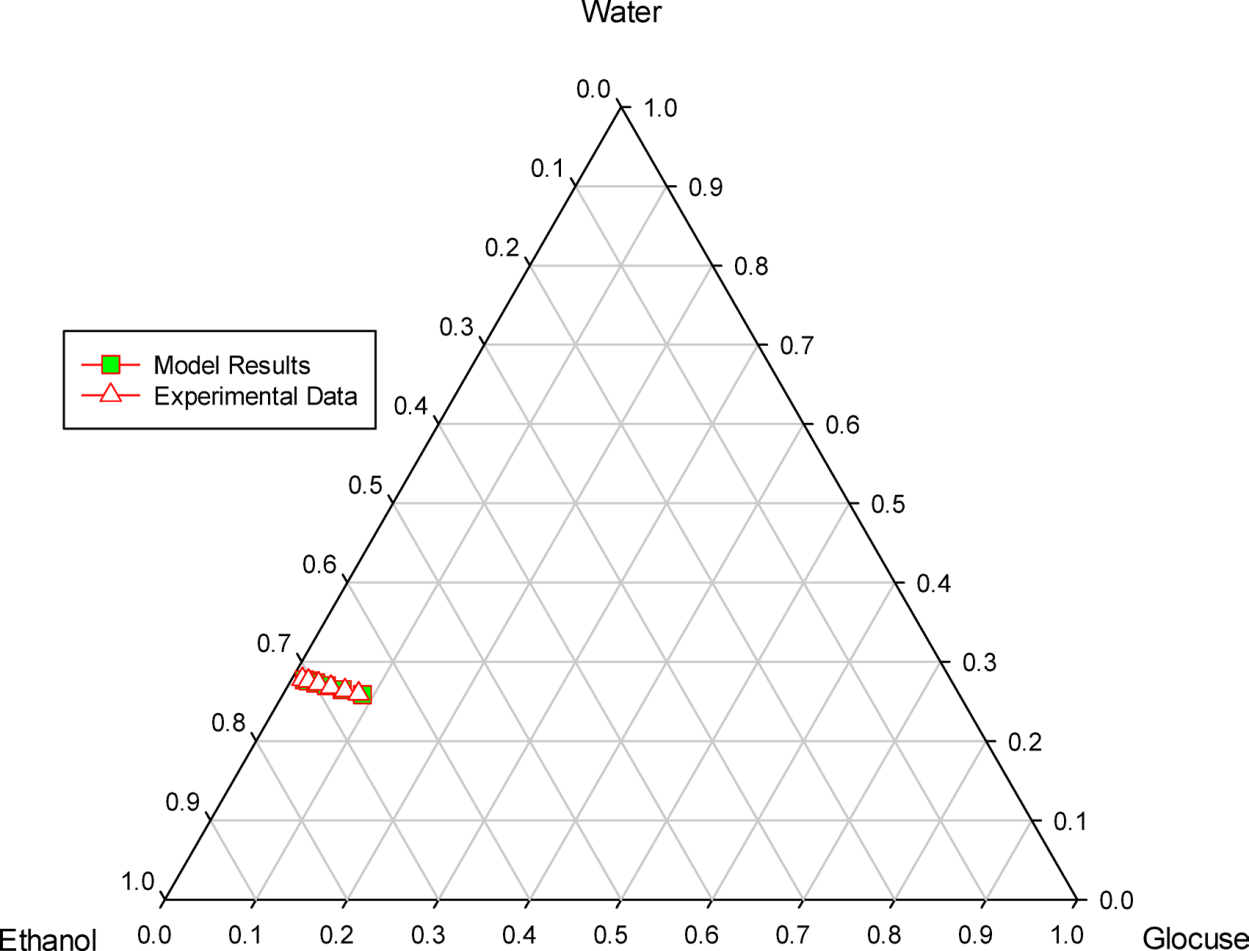
Glucose solubility in water + ethanol. Ethanol/water (50 to 50) %. (∆) Experimental data^46^and (□) model predictions.

The results show that, the PHSC model can predict the solubility of glucose in ethanol-water solvent over a wide range of temperatures satisfactory. Similar to the CO_2_-ethanol-water system, the model results are pure predictions; the results in Fig. 4 have been obtained without using any additional adjustable parameters. In Fig. 5, the triangular plot of the ternary glucose-ethanol-water system at (50/50)% of water/ethanol ratio has been predicted and compared to experimental data.

R. Dohren et al. measured the phase equilibria for the ternary system glucose-water-CO_2_ at different temperatures (323.15, 333.15 and 343.15 K) and at different pressures (10, 20 and 30 MPa)^1^. They reported the equilibrium compositions of vapor and liquid phases. In this work, the equilibrium mole fraction of components in vapor and liquid phases have been predicted and compared to experimental data. In Table 3, the PHSC EoS results have been reported at two temperatures and two pressures.

**Table 3:** Predicted and experimental equilibrium composition of ternary CO_2_-glucose-H_2_O system.

		Experimental				Predicted by PHSC EoS			
	Mole Fraction	H_2_O	CO_2_	Glucose	H_2_O	CO_2_	Glucose	ARD% x_CO2,_ y_CO2_	RMSD x_CO2,_ y_CO2_
T = 323.15 K	x	0.96382	0.01917	0.01701	0.961767	0.020195	0.0180377	0.1025	0.0010
*P* = 10 MPa	y	0.00462	0.99566	0.0000001	0.002741	0.997259	2.28233 × 10^− 12^	0.1599	0.0016
T = 323.15 K	x	0.96005	0.0230	0.01695	0.957361	0.024571	0.018068038	0.1571	0.0016
*P* = 20 MPa	y	0.00641	0.99452	0.00000054	0.004236	0.995764	2.04547 × 10^− 8^	0.1244	0.0012
T = 333.15 K	x	0.9655	0.01744	0.01706	0.959550725	0.021990917	0.018458358	0.4551	0.0046
*P* = 10 MPa	y	0.00566	0.99434	0.00000011	0.003715065	0.996284935	1.89613 × 10^− 13^	0.1945	0.0019
T = 333.15 K	x	0.96216	0.02086	0.01698	0.9537156	0.0279385	0.0183459	0.7079	0.0071
*P* = 20 MPa	y	0.000749	0.99434	0.00000010	0.0054773	0.9945227	6.59 × 10^− 9^	0.0183	0.0002

In Table 3 the AAD% refer to Average Absolute Deviation and RMSD is Root Mean Square Deviation:

17 $$\:AAD\%=\frac{100}{N}\sum\:_{i=1}^{N}\left|{X}_{i}^{exp}-{X}_{i}^{pred}\right|$$

18 $$\:RMSD=\sqrt{\frac{\sum\:_{i=1}^{N}{{(X}_{i}^{exp}-{X}_{i}^{pred})}^{2}}{N}}$$

where x^exp^ and x^pred^ refer to experimental and predicted liquid phase composition, respectively. As well, *N* refer to number of data point.

**Fig. 6:**
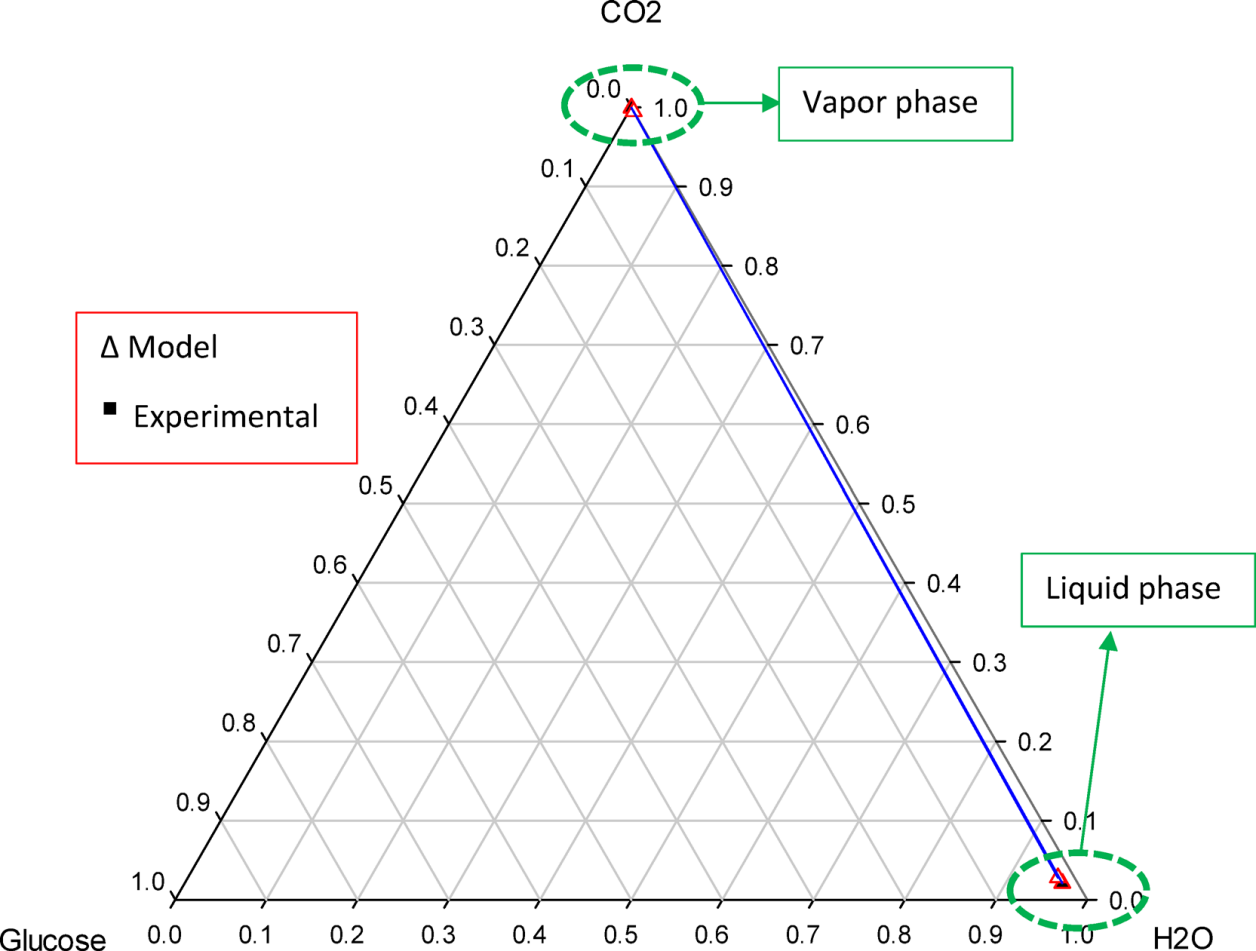
Ternary plot of glocuse-water-CO_2_ system at 333.15 K and 20 MPa.

As shown in Table 3, the PHSC EoS can predict the solubility of CO_2_ in aqueous Glucose solutions, satisfactory. The experimental and predicted solubility of CO_2_ in the liquid phase at 323.15 K and 10 MPa is obtained 0.0192 and 0.0202, respectively. The solubility of CO_2_ in the liquid phase at 323.15 K and 20 MPa is obtained 0.0245; the experimental value is 0.0230. The model deviations are increased by increasing temperature from 323.15 K to 333.15 K. For example, the solubility of CO_2_ at 333.215 K and 10 MPa was predicted 0.022, while the experimental value is about 0.0174. In the case of glucose equilibrium composition, the predicted liquid phase composition is in good agreement with experimental data. Table 3 shows that, the PHSC model cannot predict the vapor phase composition of glucose accurately. The experimental values are about 10^− 7^ to10^− 6^, while the predicted results are about 10^− 13^ to10^− 8^. It must be noted that the prediction of equilibrium compositions of sugars in the vapor phase using a thermodynamic model is difficult due to low values of sugar vapor composition (about zero). In Fig. 6, the ternary plot of glocuse-water-CO_2_ has been depicted.

As shown in Fig. 6, the ternary plot of the glucose-water-CO_2_ system has been predicted accurately.

Thermodynamic modeling of the glucose-water-CO₂ system involves capturing complex interactions among biological molecules, solvents, and gases. This is particularly relevant in biochemical processes, fermentation, environmental modeling, and chemical engineering applications. However, several challenges arise due to the system’s complexity, variables, and the limitations of existing models. Glucose, water, and CO₂ exhibit strong non-idealities, especially under high concentrations, temperature, or pressure. Capturing these interactions requires sophisticated models that go beyond ideal solution assumptions. Water forms extensive hydrogen bonds, influencing solubility and phase behavior. Modeling these interactions accurately demands advanced force fields or activity models. The significant size difference between glucose molecules and small CO₂ molecules impacts mixing, solubility, and diffusion, complicating interaction modeling. The system may involve liquid-liquid, gas-liquid, or solid-liquid phases depending on temperature and concentration. Accurately predicting phase boundaries and transitions is challenging. High glucose concentrations might lead to crystallization, adding complexity to the thermodynamic description. Processes like fermentation or carbon capture operate across broad temperature and pressure ranges; models must reliably account for these variations. The system’s thermodynamic properties depend heavily on temperature, requiring temperature-dependent parameters and models. Experimental data for glucose, CO₂ solubility, and their interactions are often limited, especially at high concentrations or non-standard conditions. Accurately calibrating activity coefficients and EoS parameters is difficult due to limited data; reliance on interpolations or assumptions may reduce accuracy. Many models require specific parameters that are not well-characterized for all systems, especially under non-ambient conditions. Therefore, modeling the glucose-water-CO₂ system thermodynamically involves tackling issues related to non-ideal interactions, phase equilibria, data scarcity, and computational complexity. Accurate predictions require sophisticated activity models, robust equations of state, comprehensive experimental data, and careful balancing of accuracy and computational feasibility. These challenges remain active areas of research in chemical engineering, biochemistry, and environmental science. In this work the PHSC EoS is used to model the VLE and SLE of sugar-containing systems. The PHSC EoS is relatively simple. It’s based on a perturbation theory around a hard-sphere chain reference fluid, making it computationally efficient. The parameters are relatively few. Offers reasonable accuracy for many systems, particularly those with relatively simple chain structures. However, it often struggles with highly associating fluids (those with strong hydrogen bonding) and systems exhibiting significant non-idealities. It extends the hard-sphere chain reference fluid to include dispersion and association contributions, making it applicable to associating fluids like alcohols and water. Requires more parameters than PHSC. The calculations are more computationally demanding. Previous research has shown that the PHSC model is simple and accurate if the model parameters are set over a wide temperature and pressure range, the objective function is appropriate, and the optimization is performed carefully. Table 3 demonstrates that the PHSC model, utilizing BIP derived from binary systems, provides good predictions for the vapor-liquid equilibrium (VLE) of the glucose-water-CO_2_ system at high pressure. The VLE calculations are strongly affected by association interactions, including cross-association between glucose and water, glucose and CO_2_, and water and CO_2_, as well as self-association of CO_2_, glucose, and water. In the next section the individual influence of each of these interaction types on the final results will be examined.

In the next section phase equilibrium calculations of the quaternary CO_2_-ethanol-water-glucose system have been studied. It must be noted that the reported results in the quaternary system are pure predictions; all results have been obtained without using any additional adjustable parameters.

### Quaternary CO_2_-ethanol-water-glucose system

The main goal of this study is the prediction of CO_2_ solubility in aqueous sugar solution in the presence of ethanol at high pressure. At low pressures, the gas phase can often be approximated as an ideal gas, simplifying the vapor-liquid equilibrium (VLE) calculations. This means that the fugacity coefficient of CO_2_ in the gas phase is close to 1. At sufficiently low partial pressures of CO_2_, Henry’s Law can be used to describe the solubility of CO_2_ in the liquid mixture. However, the Henry’s Law constant becomes a function of the liquid composition (ethanol, water, and glucose concentrations), and its determination requires experimental data. One of the main challenges in studying the quaternary CO_2_-ethanol-water-glucose system is the lack of experimental data at low pressures. In this work, the model’s performance was evaluated for the binary CO_2_-H_2_O and ternary CO_2_-H_2_O-ethanol systems at low pressures (see Sect. 3.2). The results indicated that the PHSC EoS can accurately estimate CO_2_ solubility in H_2_O and H_2_O-ethanol mixtures at low pressures. For the quaternary CO_2_-ethanol-water-glucose system at low pressures, the PHSC EoS was used to predict CO_2_ solubility in the ethanol-water-glucose solution. While EoS models are generally more complex than activity coefficient models, they remain applicable at low pressures; however, their accuracy may not be significantly superior to that of activity coefficient models, particularly if the EoS parameters are not well-optimized for low-pressure conditions.

In Sect. 3.2, the binary and ternary systems containing glucose and CO_2_ have been studied. The BIPs between all components were reported in Table 2. Similar to the ternary CO_2_-H_2_O-glucose system, the CO_2_ solubility in the H_2_O-glucose-ethanol solvent has been predicted without using any additional adjustable BIP. In the quaternary system, the increase of the glucose concentration corresponds to the increase of the water and ethanol content with rising pressure at constant feed composition^1^. R. Dohrn et al. measured the solubility of CO_2_ in H_2_O-Glucose-ethanol at 343.15 K and up to 30 MPa^1^. The equilibrium vapor and liquid compositions at 343.15 K and four pressures have been reported.

In this study, the PHSC EoS has been used to predict the solubility of CO_2_ in H_2_O-Glucose-ethanol system at different ethanol concentrations and pressures. In Table 4, the PHSC EoS results have been compared to the experimental data.

**Table 4:** Liquid phase composition of quaternary CO_2_-H_2_O-Glucose-ethanol system at 343.15 K.

Ethanol%	Pressure (MPa)	x^exp^				x^pred^				AAD% x_CO2_	RMSD x_CO2_
		H_2_O	Ethanol	CO_2_	Glucose	H_2_O	Ethanol	CO_2_	Glucose		
20% ethanol	10	0.87126	0.08811	0.02526	0.01357	0.854004	0.101433	0.031683	0.0128801	0.6423	0.0064
	15	0.7706	0.1652	0.05056	0.0136	0.759144	0.178066	0.051262	0.0115284	0.0702	0.0007
	20	0.83406	0.11742	0.03381	0.01471	0.830575	0.113284	0.04356	0.0125811	0.9750	0.0098
	30	0.81642	0.12784	0.04134	0.0144	0.804451	0.129917	0.053391	0.0122408	1.2051	0.0121
35% ethanol	10	0.60735	0.28205	0.09991	0.1069	0.644277	0.281016	0.064965	0.0097417	3.4945	0.0349
	15	0.57686	0.28403	0.12896	0.01016	0.5601	0.332874	0.09851	0.0085168	3.0450	0.0305
	20	0.68059	0.21677	0.09063	0.01201	0.664782	0.249666	0.075461	0.0100905	1.5169	0.0152
	30	0.69705	0.20929	0.08135	0.0123	0.676101	0.23296	0.080607	0.010332	0.0743	0.0007
50% ethanol	10	0.38959	0.40895	0.19462	0.00684	0.415649	0.443878	0.134158	0.0063148	6.0462	0.0605
	15	0.42010	0.39472	0.17686	0.00843	0.432872	0.413321	0.147152	0.0066552	2.9708	0.0297
	20	0.46852	0.32441	0.19883	0.00824	0.506974	0.360709	0.124556	0.007761	7.4274	0.0743
	30	0.59756	0.27428	0.11766	0.0105	0.643877	0.257062	0.089212	0.009849	2.8448	0.0284
**Average Error**										**2.5260**	**0.0253**

As shown in Table 4, the average AAD% and RMSD values for CO_2_ solubility at three ethanol concentrations and up to 30 MPa have been obtained 2.526% and 0.0253, respectively.

**Fig. 7:**
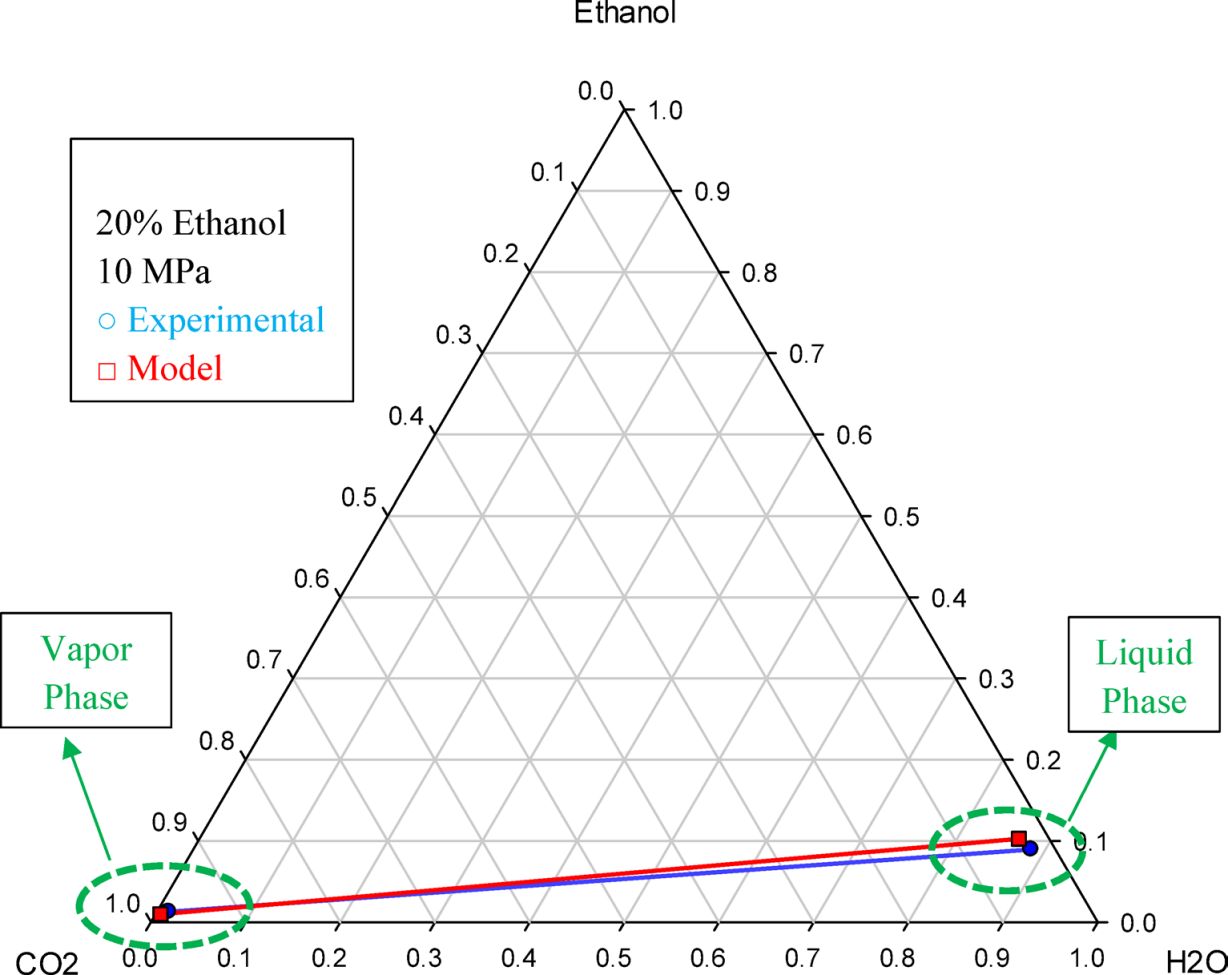
Ternary diagram of the quaternary glucose-water-ethanol-CO_2_ system at 20% ethanol, 343.15 K and 10 MPa. Figure has been depicted based on glucose-free.

**Fig. 8:**
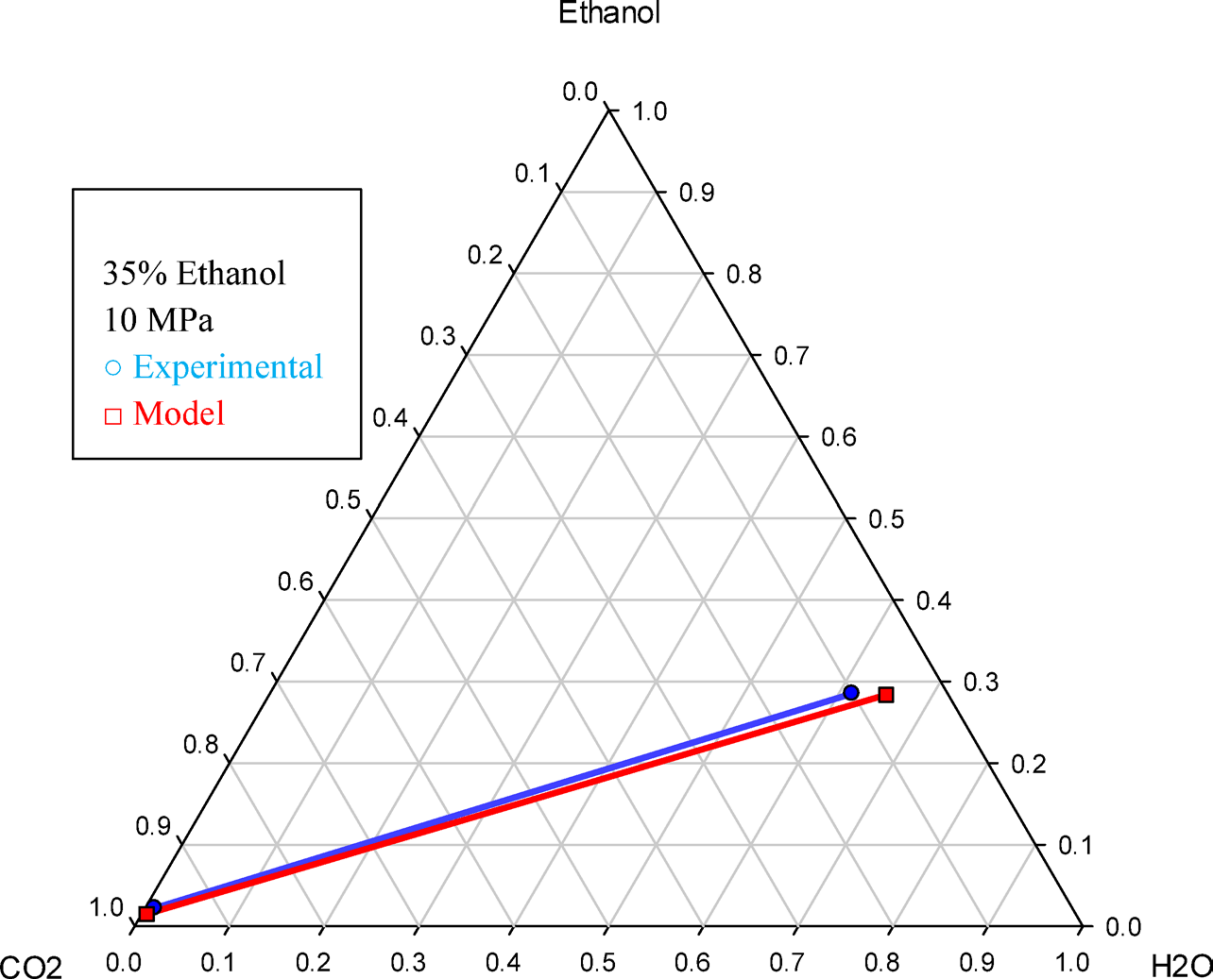
Ternary diagram of quaternary glucose-water-ethanol-CO_2_ system at 35% ethanol, 343.15 K 10 MPa. Figure haS been depicted based on glucose-free.

**Fig. 9:**
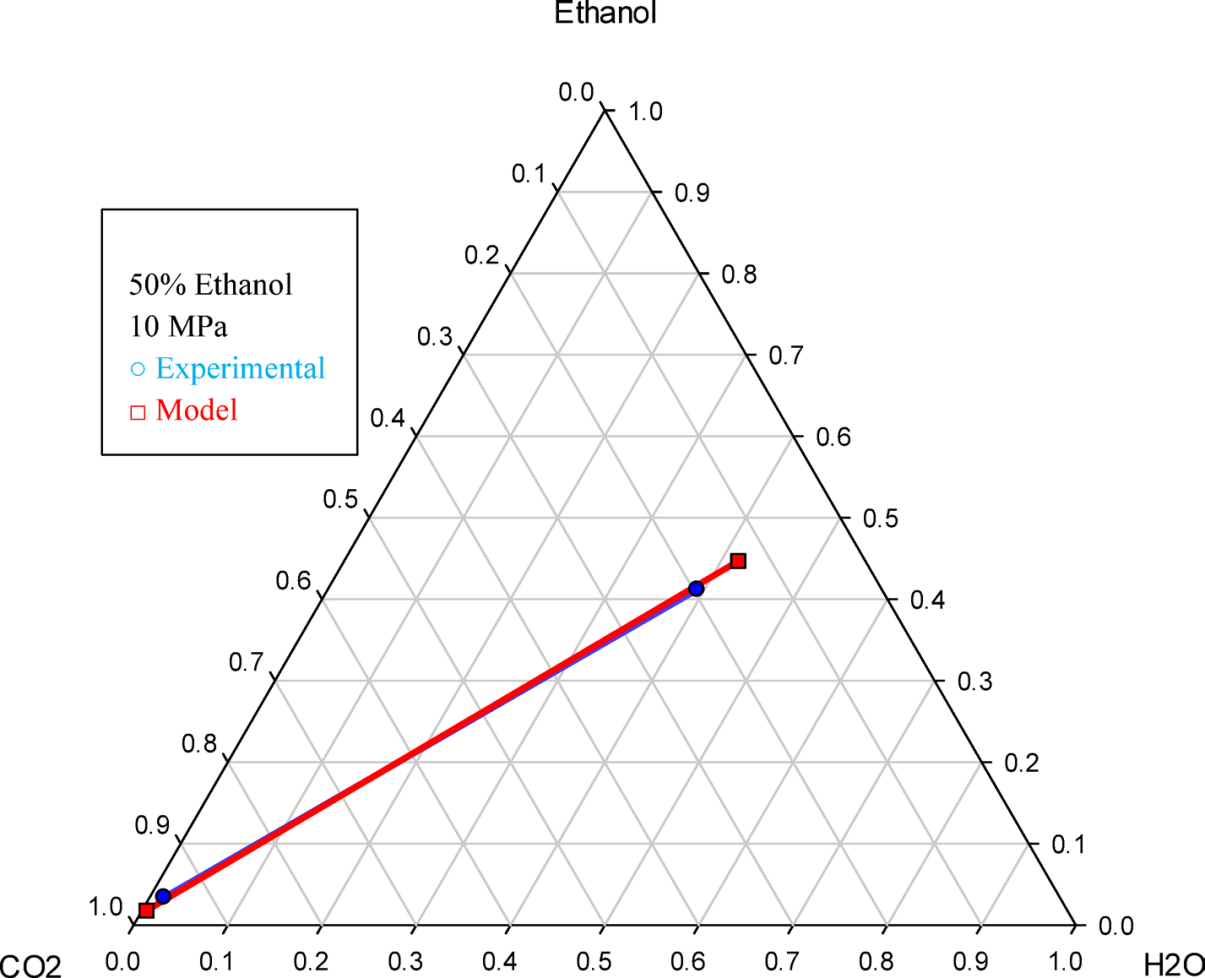
Ternary diagram of quaternary glucose-water-ethanol-CO_2_ system at 50% ethanol, 343.15 K and 10 MPa. Figure has been depicted based on Glucose-free.

In Figs. 7, 8 and 9, the triangular plot of quaternary CO_2_-H_2_O-Glucose-ethanol system at three ethanol concentration in the feed has been depicted.

As shown in Fig. 7, the PHSC model can predict the phase equilibrium calculations of the quaternary glucose-water-ethanol-CO_2_ system at 20% ethanol concentration, and 10 MPa accurately. The adjusted BIPs between all components in Table 2 have been utilized to predict the quaternary glucose-water-ethanol-CO_2_ system. It is worth noting that no additional binary interaction parameter was used in the PHSC EoS, being the results of this study purely predictive.

All results referring the solubilities of sugar are in very good agreement with experimental data. The model deviations tend to increase by increasing total pressure. In Fig. 8, the quaternary glucose-water-ethanol-CO_2_ system at 35% ethanol has been depicted.

Ternary diagram of quaternary glucose-water-ethanol-CO_2_ system at 35% ethanol has been predicted using the PHSC EoS satisfactory. The results show that, the PHSC can predict the vapor and liquid compositions of all components at 20% ethanol systems (Fig. 7) more accurately than the quaternary systems containing 35% ethanol. These results indicate that the model error increases with increasing ethanol concentration. In Fig. 9, the quaternary glucose-water-ethanol-CO_2_ system at 50% ethanol has been depicted.

According to the results of Figs. 7, 8 and 9, it is possible to confirm that the PHSC model with parametrization in Tables 1 and 2 provides good predictions for phase equilibrium calculations in quaternary systems at various ethanol concentrations. As shown in Fig. 9, the deviation between the model and experimental data in liquid and vapor phases increases by increasing ethanol concentration. In summary, the PHSC EoS can predict the solubility of CO_2_ in binary and ternary systems over a wide range of pressures and temperatures satisfactory. Different sugars (such as glucose, fructose, sucrose) can have varying effects on CO_2_ solubility due to differences in their molecular structure and how they interact with water and CO_2_ molecules. As shown in Figs. 7, 8 and 9, higher sugar concentrations generally decrease CO_2_ solubility. This is because increased sugar concentration increases the viscosity of the solution, which hinders the diffusion of CO_2_ molecules. Also, the increased density of the solution can affect the equilibrium between gas and liquid phases. As with most gases, CO_2_ solubility in sugar solutions decreases as temperature increases. Higher temperatures increase the kinetic energy of the CO_2_ molecules, making it easier for them to escape from the liquid phase. Higher CO_2_ partial pressures above the solution increase CO_2_ solubility. This relationship is described by Henry’s Law, which states that the amount of gas dissolved in a liquid is proportional to the partial pressure of the gas above the liquid. The presence of other components, such as ethanol can also affect CO_2_ solubility by altering the solution’s properties and intermolecular interactions. The presence of ethanol in the system can complicate the solubility behavior. Ethanol is miscible with water and can alter the structure of water, potentially affecting CO_2_ solubility. Interactions between glucose and ethanol molecules can further influence CO_2_ solubility. Despite the increase in model error at high ethanol concentrations, the model is well capable of modeling the solubility of carbon dioxide in the aforementioned systems up to high pressures. This indicates that the model well considers the intermolecular interactions between the components in the system.

**Fig. 10:**
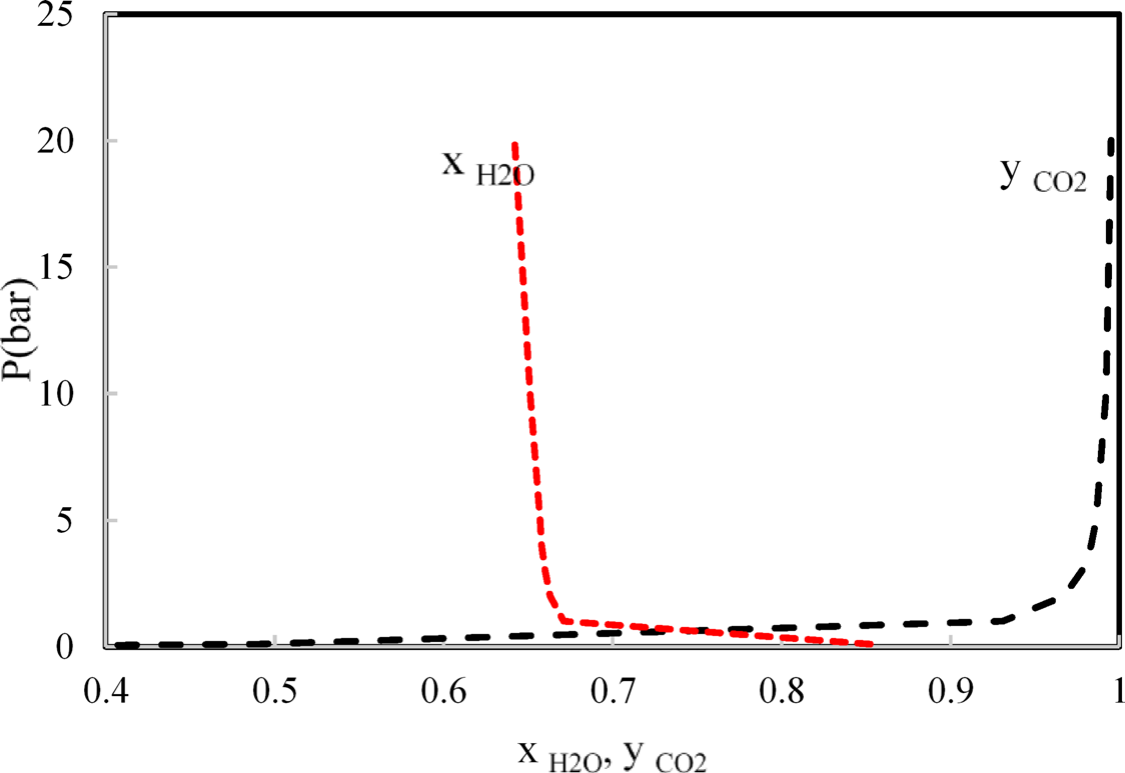
Vapor and liquid mole fraction of CO_2_ and H_2_O in quaternary glucose-water-ethanol-CO_2_ system at 50% ethanol and 298.15 K predicted using the PHSC EoS.

The effect of intermolecular interactions within the quaternary glucose-water-ethanol-CO₂ system on low-pressure CO₂ solubility calculations using an equation of state is significant. The complex interplay of hydrogen bonding, and hydrophobic effects between glucose, water, ethanol, and CO₂ profoundly influences the accuracy of predictions. Accurate models must account for these interactions to reliably predict CO₂ solubility. Failure to adequately represent these interactions can lead to substantial discrepancies between predicted and experimental values. In this work, the repulsive, dispersion, and association between all components have been considered to improve the model performance over a wide range of pressures and temperatures. In this section, the PHSC EoS has been used to predict the low-pressure solubility of CO_2_ in quaternary systems. In Fig. 10, the model prediction results at low pressures have been illustrated.

Figure 10 shows the predicted CO_2_ mole fraction in gas phase, and mole fraction of water in the liquid phase. It is important to note that, as mentioned earlier, experimental data for CO_2_ solubility in the quaternary glucose-water-ethanol-CO_2_ system do not exist at low pressures. Therefore, only the model prediction results are presented in Fig. 10. The results presented in Fig. 10 cannot be validated without experimental data, even though the PHSC model parameters were adjusted over a wide range of pressures and temperatures. The pure model parameters of water, ethanol, and CO_2_ were adjusted using experimental vapor pressure and saturated liquid density between triple point to critical point (or near critical point). The BIPs between water-ethanol, ethanol-CO_2_ and CO_2_-water were adjusted over a wide range of temperatures and pressures (including low pressures). However, without experimental data for the mixture itself at the conditions considered, the reliability of these predictions remains uncertain.

**Fig. 11:**
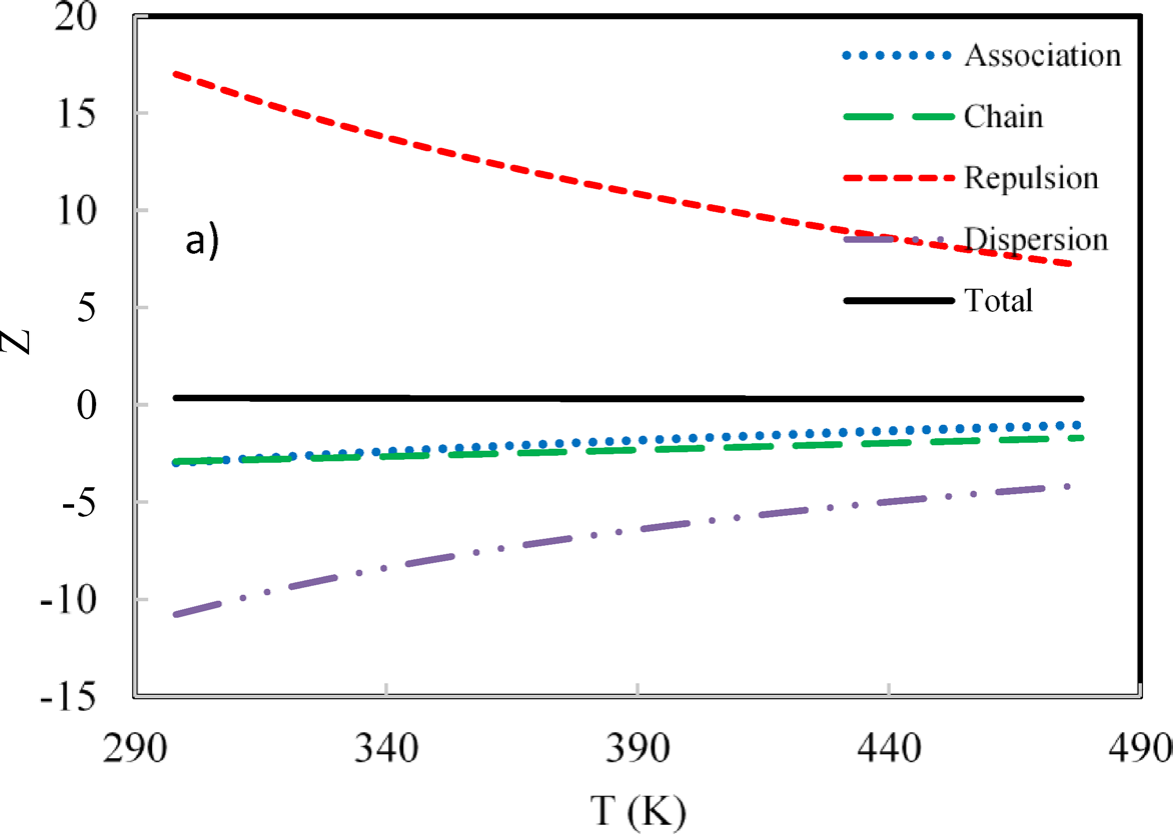
Compressibility factor vs. temperature in liquid phase for quaternary glucose-water-ethanol-CO_2_ system at 50% ethanol and 30 MPa.

**Fig. 12:**
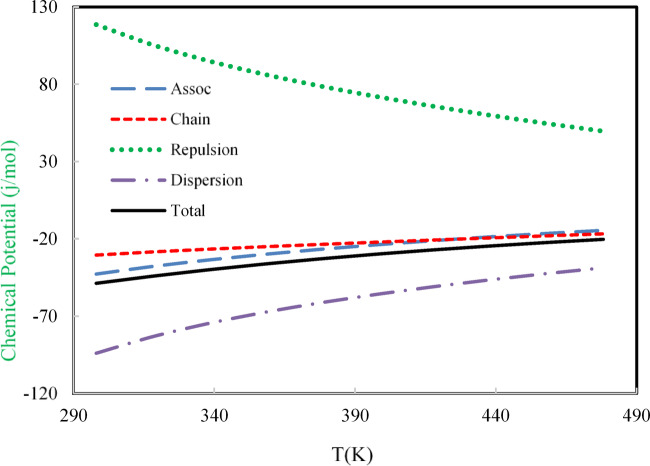
Chemical potential of PHSC EoS for glucose in liquid phase at 50% ethanol and 30 MPa.

Analyzing the chemical potential and compressibility factor within the equation of state framework is crucial for understanding phase equilibrium because it’s directly related to the driving force for phase separation. The accuracy of PHSC phase equilibrium calculations heavily relies on accurate parameters that describe the intermolecular interactions. These parameters often represent the strength and geometry of molecular interactions, and their accurate estimation from experimental data is crucial. Figures 11 and 12 show the breakdown of how the repulsion, dispersion, chain, and association forces contribute to the overall results.

In Figs. 11 and 12, the compressibility factor (Z) and chemical potentials of the PHSC EoS in quaternary glucose-water-ethanol-CO_2_ system have been depicted.

As shown in Figs. 11 and 12, the effect of all intermolecular interactions containing repulsion, dispersion, association, and chain on model performance have been investigated. The absolute values of compressibility factor of each terms tend to decrease by increasing the temperature. Similar results were obtained in the case of chemical potential of glucose in liquid phase and CO_2_ in gas phase. The association contribution shows the lower compressibility factor and chemical potential in liquid and gas phases. Dispersion and repulsion contributions show major effect on thermodynamic modeling of quaternary system. The statement indicates that:

- Association effects are less impactful on compressibility and chemical potential than other interactions (dispersion and repulsion). This is consistent with the idea that association forces, like hydrogen bonding, are stronger at lower temperatures and weaker at higher temperatures.
- Dispersion and repulsion forces are crucial for understanding the thermodynamics of the quaternary system. This suggests that these more fundamental interactions, related to intermolecular attraction and steric hindrance, are the dominant factors determining the behavior of the system overall.
- The behavior is consistent across different phases (liquid glucose and gaseous CO₂). This suggests the underlying principles governing the interactions are transferable across different states of matter.

The results highlight the relative importance of different intermolecular forces in the thermodynamics of the system, with dispersion and repulsion forces playing a dominant role. While the magnitude of association interactions is lower than that of attractive and repulsive interactions, they play a significant role in accurately modeling the phase equilibria of mixtures.

## Conclusion

In this work, the solubility of CO_2_ in ternary (CO_2_-ethanol-water) and quaternary (CO_2_-ethanol-water-glucose) systems was studied using the PHSC EoS. The main goal of this study was the prediction of CO_2_ solubility at high pressure for application to sparkling drinks. The pure sugar parameters were adjusted using the experimental Solid-Liquid equilibrium data. In the case of carbon dioxide, water, and ethanol, the pure model parameters were obtained using the vapor pressure and saturated liquid density data. Temperature-dependent BIP between all binary systems was considered to improve the model performance. CO_2_ solubility in ternary CO_2_-ethanol-water systems was predicted without using any additional adjustable parameters. The results show that, the PHSC EoS can predict the equilibrium composition of vapor and liquid phases in ternary CO_2_-ethanol-water systems satisfactory. The PHSC model can predict the solubility of glucose in the ethanol-water solvent over a wide range of temperatures satisfactory. Similar to the CO_2_-ethanol-water system, the model results were pure predictions. In the case of CO_2_-glucose-water system, CO_2_ solubility was predicted up to 20 MPa using the PHSC EoS satisfactory. According to the PHSC results in the quaternary CO_2_-ethanol-water-glucose system, it is possible to confirm that the PHSC model provides good predictions for phase equilibrium calculations at various ethanol concentrations. The results show that the model deviations tend to increase by increasing ethanol concentrations. The proposed model can be used to predict the CO_2_ solubility in sparkling drinks at various pressures and temperatures. The effect of alcohol concentration, pressure, and temperature on sparkling drink quality can be studied using the PHSC EoS. The proposed methodology can be extended to various sparkling drinks or juices with different sugars.

## Data Availability

All data generated or analysed during this study are included in this published article.
